# mTOR activation triggers proinflammatory expansion of IL-4-producing and necrosis-prone double-negative T cells, precedes flares, and serves as target for treatment in patients with systemic lupus erythematosus

**DOI:** 10.1186/ar4641

**Published:** 2014-09-18

**Authors:** Zhi-Wei Lai, Rebecca Borsuk, Ashwini Shadakshari, Jianghong Yu, Maha Dawood, Ricardo Garcia, Lisa Francis, Hajra Tily, Adam Bartos, Stephen V Faraone, Paul Phillips, Andras Perl

**Affiliations:** 1 State University of New York, Upstate Medical University, College of Medicine, Syracuse, NY, USA

## Background

Oxidative stress is increased in systemic lupus erythematosus (SLE), and it contributes to immune system dysregulation and fatal comorbidities. Mitochondrial dysfunction in T cells promotes the release of highly diffusible inflammatory lipid hydroperoxides, which spread oxidative stress to other intracellular organelles and through the bloodstream. In T cells from patients with SLE and animal models of the disease, glutathione, the main intracellular antioxidant, is depleted and the mechanistic target of rapamycin (mTOR), serine/threonine protein kinase, undergoes redox-dependent activation. In turn, reversal of glutathione depletion by application of its amino acid precursor, *N*-acetylcysteine, blocks mTOR activation and improves disease activity in lupus-prone mice and patients with SLE. While mTOR has been also recognized as an effector of T-cell lineage development, its role in autoimmunity and disease activation remain unclear.

## Methods

Here, we prospectively examined mitochondrial dysfunction and mTOR in PBL relative to SLEDAI and BILAG disease activity indices during 274 visits of 59 patients and 54 healthy subjects matched for each patient blood donation. A total of 212 metabolic biomarkers and traditional biomarkers, anti-DNA, C3, and C4, were evaluated by partial least-square discriminant analysis (PLS-DA). False discovery rate (FDR) *P *values were determined for each contributing biomarker and considered significant at *P *< 0.000236 with correction for multiple comparisons (0.05/212). Medication use was compared between patient groups exhibiting flare and remission with chi-square and Fischer's exact tests.

## Results

PLS-DA identified 15 of 212 parameters that accounted for 70.2% of the total variance and discriminated lupus and control samples (*P *< 0.0005); increased mitochondrial mass of CD3^+^/CD4^-^/CD8^- ^double-negative (DN) T cells (*P *= 1.1 × 10^-22^) and FoxP3 depletion in CD4^+^/CD25^+ ^T cells were top contributors (*P *= 6.7 × 10^-7^). Prominent necrosis and mTOR activation were noted in DN T cells during 15 visits characterized by flares (SLEDAI increase ≥4) relative to 61 visits of remission (SLEDAI decrease ≥4). mTOR activation in DN T cells was also noted at preflare visits of SLE patients relative to those of stable disease or healthy controls. DN lupus T cells showed increased production of IL-4, which correlated with depletion of CD25^+^/CD19^+ ^B cells. Rapamycin treatment *in vivo *reduced SLEDAI and BILAG, blocked the IL-4 production and necrosis of DN T cells, increased the expression of FoxP3 in CD25^+^/CD4^+^T cells, and expanded CD25^+^/CD19^+ ^B cells.

## Conclusions

These results identify mTOR activation to be a trigger of IL-4 production and necrotic death of DN T cells, predictor of disease flares, and effective target for treatment in patients with SLE.

